# Mandibular Dentigerous Cyst in a 10-Year-Old Child

**DOI:** 10.5005/jp-journals-10005-1378

**Published:** 2016-09-27

**Authors:** Bindu Bhardwaj, Sunil Sharma, Punit Chitlangia, Prateek Agarwal, Amit Bhamboo, Komal Rastogi

**Affiliations:** 1 Professor, Department of Oral Surgery, Mahatma Gandhi Dental College and Hospital, Jaipur, Rajasthan, India; 2 Professor and Head, Department of Oral Surgery, Mahatma Gandhi Dental College and Hospital, Jaipur, Rajasthan, India; 3 Reader, Department of Oral Surgery, Mahatma Gandhi Dental College and Hospital, Jaipur, Rajasthan, India; 4 Senior Lecturer, Department of Oral Surgery, Mahatma Gandhi Dental College and Hospital, Jaipur, Rajasthan, India; 5 Senior Lecturer, Department of Oral Surgery, Mahatma Gandhi Dental College and Hospital, Jaipur, Rajasthan, India; 6 Private Consultant, Department of Oral Surgery, Rajasthan India

**Keywords:** Dentigerous cyst, Enucleation, Marsupialization.

## Abstract

Dentigerous cyst is the most common odontogenic cyst. It is characterized by a unilocular radiolucent lesion that encloses permanent tooth buds or, under certain circumstances, displaced tooth buds. Buccal bony expansion and a missing tooth is the most common clinical feature. Various treatment modalities have been mentioned in the literature for management of dentigerous cysts. This article presents a left mandibular dentigerous cyst in a 10-year-old boy. Marsupialization was the treatment of choice and a denturelike space maintainer was provided. Long-term follow-up revealed good healing of the bony lesion.

**How to cite this article:**

Bhardwaj B, Sharma S, Chitlangia P, Agarwal P, Bhamboo A, Rastogi K. Mandibular Dentigerous Cyst in a 10-Year-Old Child. Int J Clin Pediatr Dent 2016;9(3): 281-284.

## INTRODUCTION

Dentigerous cyst is the most common odontogenic cyst. It is associated with the crown of an unerupted tooth. The cyst cavity is lined with reduced enamel epithelium derived from the tooth-forming organ.^1^ The cyst is a well-defined radiolucent lesion with fluid accumulation between the epithelium and tooth crown. The clinical findings are cortical bone expansion, adjacent permanent tooth bud displacement, and root dilacerations. The developmental type is usually found in the late 2nd and 3rd decades. It occurs in mature teeth, generally without inflammation. The inflammatory type is found in the 1st and early 2nd decades. It usually occurs in a nonvital immature deciduous tooth.^2-7^

## CASE REPORT

**Fig. 1:**
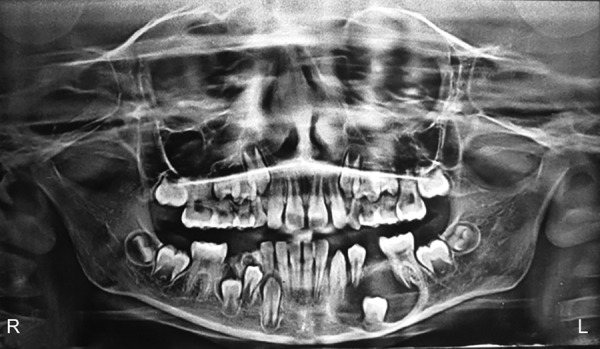
Orthopantomogram (OPG) showed a large radiolucent lesion with erupting 2nd premolar in left mandibular body region

**Fig. 2:**
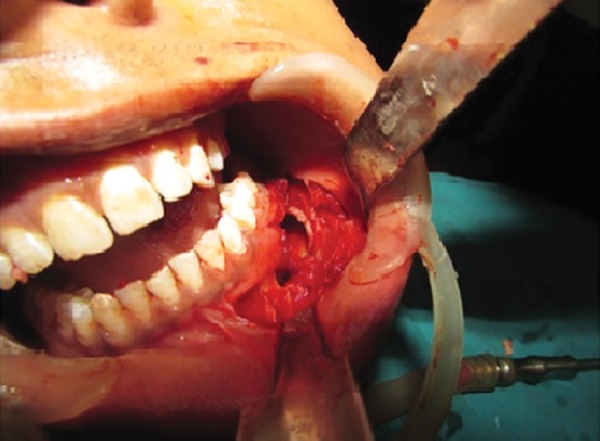
Marsupialized cystic cavity

A 10-year-old boy was referred to the department of oral and maxillofacial surgery for evaluation of pain and swelling in the left mandibular vestibule. Physical examination revealed a hard submucosal mass with buccal cortical plate expansion in region of second premolar. A panoramic radiograph showed a well-defined radiolucent lesion, measuring approximately 3 × 2 cm in diameter and including the crowns of the unerupted mandibular left 2nd premolar. The root of the adjacent canine, 1st premolar, and 1st molar were also involved in the lesion. The root apices of these teeth were still not closed. No signs of root resorption were present (Fig. 1). Considering the age of the patient and vicinity to the lower border of mandible, marsupialization of the cystic cavity with preservation of all the involved teeth was planned. Intraoperatively (Fig. 2) due to excessive mobility of the 2nd premolar, the tooth could not be preserved. Postoperatively, space maintainer was provided to prevent the tilting of the 1st premolar and molar into the defect.

**Fig. 3:**
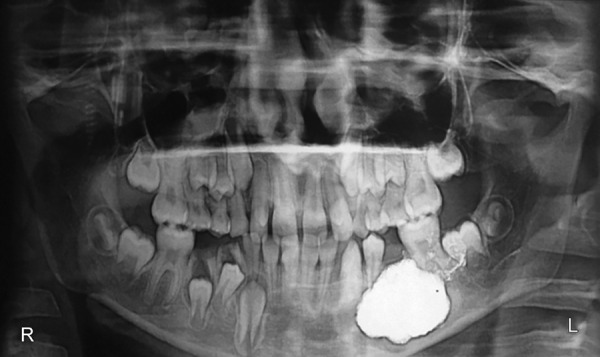
Immediate postoperative view with iodoform packing

**Fig. 4:**
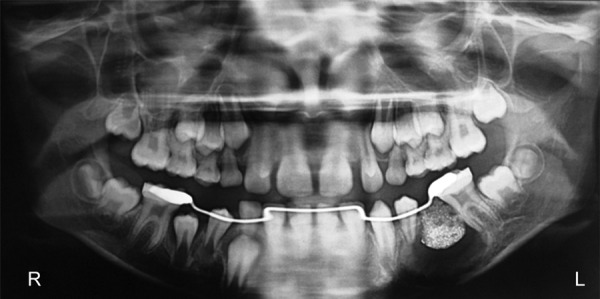
Three-month postoperative image showed shrinkage of cystic cavity and a space maintainer

**Fig. 5:**
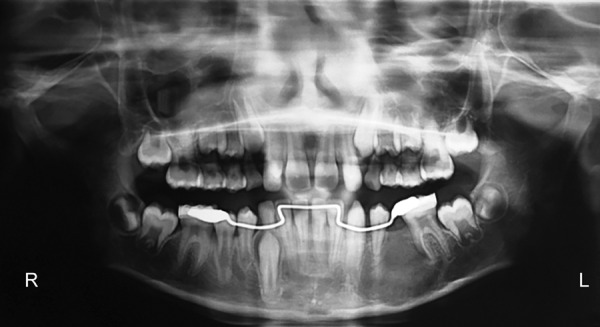
Six-month postoperative image showed complete regression of the lesion

**Fig. 6:**
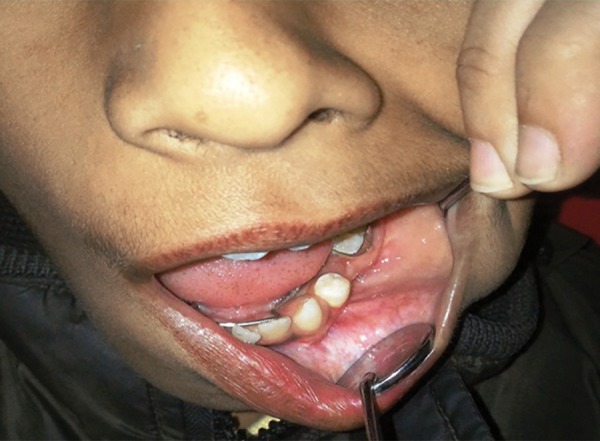
Follow-up intraoral photograph showed complete healing of the defect

The patient was treated with extraction of the 2nd premolar, marsupialization of the cystic lesion (Fig. 3) by creating a window of 1 × 1 cm in the lower left buccal vestibule the cystic cavity, and placement of space maintainer (Fig. 4). The cystic lesion was sent for histo-pathologic evaluation. Histologically, the epithelial lining with hyperplastic rete ridges was thick. The collagenized fibrous cyst capsule showed a diffuse chronic inflammatory cellular infiltration. According to the histologic diagnosis, the bony lesion was a dentigerous cyst. The marsupialized wound was sutured and packed with iodoform gauze for a week. The patient was instructed to clean the wound with distilled water from a syringe after each meal. A 6-month postoperative radiograph showed regression (Fig. 5) and disappearance of the cystic lesion (Fig. 6).

## DISCUSSION

Dentigerous cyst is associated with crown of unerupted teeth.^8^ It can cause cortical plate expansion and result in facial asymmetry.^9^ A unilocular well-defined radio-lucency lesion and asymptomatic lesion characterized on radiographic examinations.^2^ Other radiolucent lesion, such as radicular cyst, odontogenic keratocyst, ameloblastoma, and odontoma should be differentially diagnosed with dentigerous cyst.^10^

The pathogenesis of dentigerous cyst is by the accumulation of fluid either between the reduced enamel epithelium and the enamel or between the layers of the enamel organ. This fluid accumulation might be a result of pressure exerted by a potentially erupting tooth on the follicle, which obstructs the venous outflow and induces serum transudation across the capillary wall.^2,11^

In 1928, Bloch-Jorgensen, in a series of 22 cases of dentigerous cysts, reported a diseased deciduous tooth or a remnant found in direct contact with the cyst wall. This finding has also been pointed out by Azaz and Shteyer,^12^ Shaw et al,^13^ Benn and Altini,^2^ Bando and Nagayama,^14^ and Aguiló and Gandía.^15^

Sharp and Helsper^16^ has demonstrated that dentigerous cyst is caused by an alteration of the normal development of the tooth germ related to mechanical obstruction or an eruptive deviation.^16^

Various treatment modalities include complete enu-cleation and marsupialization. The choice of treatment depends on various factors, such as age of the patient, location of the cyst, tooth position in relation to the cyst, and the degree of the axial inclination and root formation. If the cyst is associated with a supernumerary tooth, complete enucleation of the cyst along with extraction of the tooth may be the first choice.^17-20^ If preservation of the displaced teeth is desirable, marsupialization is a rather conservative treatment option.^21-26^ Marsupialization is the conversion of a cyst into a pouch by suturing the cyst lining to the oral mucosa. This method has fewer complications than enucleation regarding the preservation of important anatomical structures and developing permanent tooth germs.

The treatment of dentigerous cyst is usually surgi-cal,^2,11-16,27-34^ which may consist of enucleation and extraction of the teeth embedded in it or affected by it. In very large cysts, an initial phase of marsupialization of the lesion to the oral cavity followed by enucleation is also recommended. Although Kaban^34^ only mentions occasional tooth-saving as a possibility, he does state that thorough enucleation should never be compromised.

However, there are various single case reports where the cyst was opened to the oral cavity and a stent - either a rubber tube, a removable device, or a gauze packing -was used to keep the opening patent.^35-38^

Children have greater capacity to regenerate the bony structure compared to adults; moreover, teeth with open apices have a great eruptive potential.^39^ These factors are significant in cases of large dentigerous cyst in children and presents a better prognosis for the teeth involved in the lesion.

## CONCLUSION

The choice of treatment for dentigerous cyst is ruled by various factors, such as age of patient, location and size of the cyst, tooth position in relation to the cyst, proximity to the vital structures, and degree of the axial inclination of the tooth and its root formation. Marsupialization stands as a favorable or preferred treatment modality in young patients.
